# Planteome 2024 Update: Reference Ontologies and Knowledgebase for Plant Biology

**DOI:** 10.1093/nar/gkad1028

**Published:** 2023-12-06

**Authors:** Laurel Cooper, Justin Elser, Marie-Angelique Laporte, Elizabeth Arnaud, Pankaj Jaiswal

**Affiliations:** Department of Botany and Plant Pathology, Oregon State University, Corvallis, OR 97331, USA; Department of Botany and Plant Pathology, Oregon State University, Corvallis, OR 97331, USA; Digital Inclusion, Biodiversity International, 34397 Montpellier, France; Digital Inclusion, Biodiversity International, 34397 Montpellier, France; Department of Botany and Plant Pathology, Oregon State University, Corvallis, OR 97331, USA

## Abstract

The Planteome project (https://planteome.org/) provides a suite of reference and crop-specific ontologies and an integrated knowledgebase of plant genomics data. The plant genomics data in the Planteome has been obtained through manual and automated curation and sourced from more than 40 partner databases and resources. Here, we report on updates to the Planteome reference ontologies, namely, the Plant Ontology (PO), Trait Ontology (TO), the Plant Experimental Conditions Ontology (PECO), and integration of species/crop-specific vocabularies from our partners, the Crop Ontology (CO) into the TO ontology graph. Currently, 11 CO vocabularies are integrated into the Planteome with the addition of yam, sorghum, and potato since 2018. In addition, the size of the annotation database has increased by 34%, and the number of bioentities (genes, proteins, etc.) from 125 plant taxa has increased by 72%. We developed new tools to facilitate user requests and improvements to the CO vocabularies, and to allow fast searching and browsing of PO terms and definitions. These enhancements and future changes to automate the TO-CO mappings and knowledge discovery tools ensure that the Planteome will continue to be a valuable resource for plant biology.

## Graphical Abstract

**Graphical Abstract:**
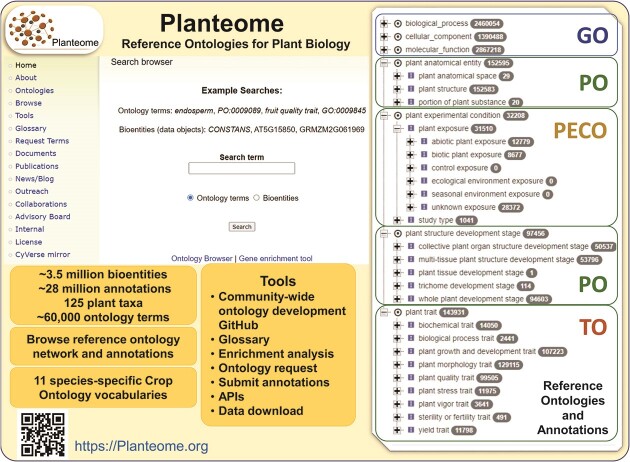

## Introduction

The Planteome Project (https://planteome.org/) provides a suite of reference and crop-specific ontologies and an integrated knowledgebase of annotated plant genomic data. Three of the reference ontologies, the Plant Ontology (PO) (1–3), the Plant Trait Ontology (TO) (4), and the Plant Experimental Conditions Ontology (PECO) (5), have been developed and are maintained in-house by the Planteome team. At the same time, the Gene Ontology (GO) (6), the Phenotype and Trait Qualities Ontology (PATO) and the NCBI Taxonomy (7) are imported for extended cross-references and standardized annotation of omics and genetics data as needed. The crop-specific ontologies developed by our partners at the Crop Ontology (CO; https://cropontology.org/) are mapped to the TO for integration into the Planteome. The Planteome conforms to the Findable, Accessible, Interoperable, and Re-usable (FAIR) data policy standards (8), and in a broadly collaborative effort, it hosts plant genomics data from more than 40 databases and resources (Table 1).

**Table 1.** Summary of Planteome updates

	Version 2.0	Version 3.0	Version 4.0	Version 5.0
Release Date	February 2017	September 2018	October 2020	July 2023
Ontology terms (excluding obsoletes)	51,874	59,189	59,827	59,485
CO mapped to TO	8	10	11	11
Total annotations	21,206,117	21,400,874	19,838,934	28,399,673
Total bioentities	2,000,665	2,057,205	3,060 530	3,449,543
Total taxa	94	95	124	125
Annotation data sources	21	22	41	42
**Planteome Reference Ontology Release Version DOI#**				
Plant Ontology (PO)	10.5281/zenodo.268024	10.5281/zenodo.1435100	10.5281/zenodo.3993787	10.5281/zenodo.8145192
Trait Ontology (TO)	10.5281/zenodo.268033	10.5281/zenodo.1435098	10.5281/zenodo.4086182	10.5281/zenodo.8161339
Plant Experimental Conditions Ontology (PECO)	10.5281/zenodo.1069485	10.5281/zenodo.1345219	10.5281/zenodo.3994925	10.5281/zenodo.8184309

Three Planteome database releases since 2017 include revisions to existing ontology terms, the addition of new ontology terms, and annotations from new data sources and taxa. The number of plant taxa covered has increased along with the total number of bioentities (genes, proteins, QTLs, gene models, etc.).

## Updates to the Planteome knowledgebase

Since the publication of the original Planteome paper in 2018 (5), many updates and improvements have occurred to the reference and species-specific ontologies and the annotation database. We have also created new tools for the community to access and interact with the Planteome ontologies and database. At the time of the previous publication, the Planteome platform was in the second full release, and we have recently released the Planteome Version 5.0 in July 2023. A summary is available in Table 1, and full details of the changes reported in each release are accessible from the release notes at https://planteome.org/documents.

### Changes to the reference ontologies

All the reference ontologies are managed through the Planteome GitHub organization (https://github.com/Planteome), where changes to the ontologies and metadata are under version control. Each ontology is stored in a separate repository and is associated with an issue tracker for comments, new term requests and suggested revisions. Each of the ontologies undergoes a release through its respective GitHub repository, and since 2016, the release versions of the Planteome reference ontologies are assigned a DOI through Zenodo (https://zenodo.org) (Table 1). The release process updates the files at the stable URLs, which are available through various ontology look-up sites and libraries, for example, for the Trait Ontology (TO) at the OBO Foundry (http://obofoundry.org/ontology/to.html) and the EBI-OLS (https://www.ebi.ac.uk/ols4/ontologies/to). The DOI for the most recent ontology version is displayed as a badge on the README file in the respective GitHub repository. The Planteome database releases incorporate the latest versions of the reference and species-specific ontologies, along with the updated annotation database. These are loaded together on the Planteome ontology browser on the Planteome website: https://planteome.org/. During the Planteome version 5.0 preparation, there was a net decrease of 342 ontology terms (Table 1). This change primarily results from removing the GO terms that were either marked obsolete (not to be used) or merged into another term (https://geneontology.org/stats.html).

- **Plant Ontology**

The PO describes plant anatomical structures and their developmental stages. Significant revisions include 22 new terms to describe different types of inflorescences, three new terms, and two revised ones to describe different types of pedicels, as well as new terms were added to describe parts of the metaxylem vessel, the endodermis cell, and the cortex-endodermis initial cell.

Extensive revisions included updates to the inflorescence, branch, and inflorescence axis terms, stolon, spikelet and its child terms, paraclade, plant ovule integument, and root initial cell terms. We have added ‘plant’ to the name of all ‘epidermis’ terms: plant epidermis, plant epidermal cell, and plant epidermal initial cell to avoid confusion with the animal epidermis, etc., during annotation, and updated the definitions of 45 child terms.

Other changes to the PO include adding a taxon constraint at the root level, changing the obo namespace from ‘plant anatomy’ to ‘anatomical entity,’ and updating the database cross-references and links to the issue trackers on GitHub.

- **Plant Trait Ontology**

The TO consists of nine branches describing the trait classes: biochemical, biological process, plant growth and development, plant morphology, plant quality, plant stress, plant vigor, sterility or fertility and yield. Definitions of many of the terms were revised or added if missing. Design patterns were created for almost half the terms in the ontology.

Significant additions and updates to the *plant morphology trait* (TO:0000017) branch include 41 new terms for various morphological traits of different fruit types, including grain (caryopsis fruit), and capsule fruits, such as those found in poppies and cotton. In addition, 32 new trait ontology terms were added to describe fresh and dry weights of various other plant structures. We added eight new terms for inflorescence traits, with four specifically related to the characters of a panicle inflorescence. Fifteen new terms were created to describe leaflet traits and five for bulb morphology. Ten new traits of roots and root systems were created, including subterranean shoot axis tubers, such as in potatoes and yams.

In the *biochemical trait* (TO:0000277) branch, 49 new terms for mineral contents in shoot and root systems were added, along with another 53 new terms for potassium, nitrogen, phosphorus, and carbon contents in other plant structures. Two new terms were added for fatty acids, linolenic acid, and palmitic acid contents.

Three new traits were added in the *plant quality trait* (TO:0000597) branch for traits associated with processing, cooking, and brewing qualities, and two new terms to describe fruit shattering in the *plant growth and development trait* (TO:0000357) branch. Modifications to the *plant stress trait* (TO:0000164) branch include new biotic stress traits and changes to align with the Plant Experimental Conditions Ontology (PECO) and the Plant Stress Ontology (PSO), which is under development. A new *yield trait* (TO:0000371) parent term was added to describe the yield of legume fruits.

- **Plant Experimental Conditions Ontology**

The Plant Experimental Conditions Ontology (PECO) describes exposures or treatments to plant or plant part(s). The PECO was originally the Plant Environment Ontology (EO) but was renamed in October 2017 to avoid confusion with the Environment Ontology (ENVO) (9).

Updates to the PECO include creating new terms to describe exposures to potassium fertilizer, potassium chloride salt, nematicide, three terms for organic soil amendments, aeroponic plant growth media and two plant pathogens. Other changes include the addition of design patterns to automate imports of terms from other reference ontologies, such as Chemical Entities of Biological Interest (10).

### Updates to Planteome annotation data

The Planteome annotation database is stored in a custom version of an Apache SOLR index originally created by the Gene Ontology called GOLR (11). This database stores the ontology terms, relationships and annotations to biological entities (bioentities) such as proteins, genes, gene models, transcripts, quantitative trait loci (QTLs), etc. The bioentity types, or data objects, are produced and named by the source databases or resources using their terminology. Planteome provides database cross-reference links back to the source database. We have preserved the nomenclature to enable establishing cross-references and interoperability. The annotations, or links between the ontology terms and the bioentities, are contributed by collaborators and also generated in-house. Since Version 2.0 of the project in 2017, the number of annotations has increased by 34% from 21,206,117 to 28,399,673, and bioentities by 72% from 2,000,665 to 3,449,543.

Between the release of Planteome version 3 in September 2018 and version 4.0 in October 2020, we updated the annotation data files in the Planteome. We removed a large number of transcript isoforms based on the gene/transcript ID information provided by the source databases. Our goal in Planteome is to provide annotations from sequenced genomes at the bioentity ‘gene’ level. Ideally, a single canonical (longest and structurally complete) transcript form represents a gene bioentity. Additionally, new versions of gene sequences with better evidence support and homology-deriving software ideally result in better and more stringent scores, thus reducing the counts. We revised some annotations due to GO vocabulary revisions, updates to the original *Arabidopsis thaliana* annotations, and their projections to the homologs with taxon/species-specific restrictions.

The bulk of the increase in bioentities was in the update from Version 3.0 to 4.0 (October 2020), where annotations from 19 new source databases were added (Table 1). In addition, 25 new taxa were added to the pipelines where annotations were generated using both InterProScan (IPRScan) (12) and by orthology to *A. thaliana* for GO annotations, maintained by TAIR (https://www.arabidopsis.org/; (13). The source GO annotations from TAIR are first updated at the Gene Ontology Consortium site, followed by updates on the Planteome. In the latest release, there were 99 species for whom the InterProScan-based GO annotations were supplemented with predicted assignments via gene homology to *A. thaliana* genes with experimentally validated GO annotations.

We updated the IPRScan-based GO annotations for new species data and to integrate updates or changes to the InterProScan program. A total of 101 species were annotated in this way. Annotations were also checked and changed if they were annotated to obsolete ontology terms, had incorrect ontology aspects, changed external references, etc. A script was developed (available on GitHub) to validate annotations and check quality for issues.

Planteome Version 4.0 included the addition of annotations to the flowering dogwood (*Cornus florida*) genome. In the latest release, Version 5.0, we added 7,675 newly curated Sorghum QTLs (https://aussorgm.org.au/sorghum-qtl-atlas/; (14) annotated to TO terms, and 4,431 QTLs from SoyBase (https://www.soybase.org/; (15) annotated to terms from TO and PO.

### Mapping Crop Ontologies and integration into the Planteome Knowledgebase

**Figure 1.**
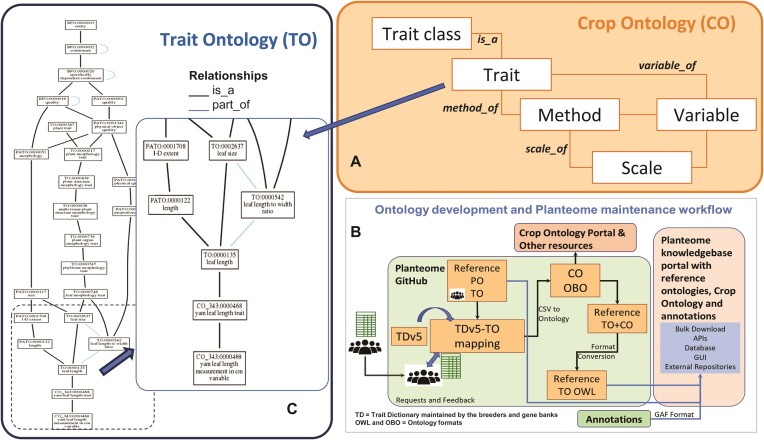
Crop Ontology Schema and the Workflow for Integration into the Planteome. (**A**) a variable is linked to a trait, method and scale in the Crop Ontology schema. (**B**) A Crop Ontology trait dictionary (TD) is maintained on the Planteome GitHub, where it is converted from the CSV format to OBO format and mapped to the Trait Ontology (TO). (**C**) A CO trait term associated with a variable is integrated into the TO hierarchy based on the mappings.

An important aspect of the Planteome is to serve as an integrative layer between in-house and other reference ontologies and the application vocabularies (Figure 1). The Crop Ontology (CO) project (https://cropontology.org/) manages a set of crop-specific ontologies used by plant breeders in their field books, ensuring that phenotype-trait data is captured using standardized ontologies, and maintains harmonization, reusability, and interoperability (16,17). The CO project is widely adopted and has become an essential component of plant breeding databases such as the Breeding Management System of the Integrated Breeding Platform (https://www.integratedbreeding.net/) and the Breedbase (https://breedbase.org/) (18). The CO vocabularies are developed as trait dictionaries (TDs) by plant breeders and curators from CGIAR centers and collaborators worldwide. The TD standards follow their Guidelines v. 2.1 (19) and provide a common vocabulary and structure for describing phenotype-trait variables and scores that breeders are recording in the field. The crop/species-specific TD template v. 5.2, as specified by the CGIAR organization, is currently used. Each species-specific CO vocabulary is assigned a unique identifier prefix (CO_DDD), where the DDD is defined by the Crop Code (https://cropontology.org/page/CropCodes) (Table 2). The TDs consist of trait classes, traits, variables, methods, and scales specific to the individual crops and are saved as comma-separated values (CSV) files. The variables (observations) are used to annotate data precisely as they link to a trait (what is measured), a method (how is it measured) and a scale (how the data is reported or scored, using a qualitative or quantitative scale or a unit of measurement) (Figure 1A).

**Table 2.** Summary of Crop Ontology (CO) trait mappings to the reference Plant Trait Ontology (TO)

Crop name	Crop code	Mapping method	Trait #	When added	Exact matches	Subclass (broad/narrow) matches	Total mapped
Rice (*Oryza sativa*)	CO_320	SSSOM	267	V 2.0	129	26	155
Wheat (*Triticum aestivum)*	CO_321	DP	322	V 2.0	38	215	253
Maize (*Zea mays*)	CO_322	DP	197	V 2.0	146	33	179
Sweet potato (*Ipomoea batatas*)	CO_331	DP	193	V 2.0	13	161	174
Cassava (*Manihot esculenta)*	CO_334	DP	167	V 2.0	39	111	150
Soybean (*Glycine max*)	CO_336	DP	82	V 2.0	61	18	79
Lentil (*Lens culinaris*)	CO_339	DP	65	V 2.0	23	42	65
Pigeon pea (*Cajanus cajan*)	CO_341	DP	61	V 2.0	21	34	55
Sorghum (*Sorghum bicolor)*	CO_324	SSSOM	121	V 3.0	47	74	121
Yam (*Dioscorea sp*.)	CO_343	DP	154	V 3.0	24	125	149
Potato (Solanum tuberosum)	CO_330	SSSOM	149	V 4.0	17	93	110

Eleven crop ontologies were mapped to the TO, using either the Design Pattern (DP) approach or the Simple Standard for Sharing Ontology Mappings (SSSOM) approach.

The CSV format TDs are converted to an OBO format (20) ontology file, where the mapping to the TO is carried out (Figure 1B). Since the TO already has an ontology graph of classes and subclasses, the categorical trait classes described in the TD are ignored. As different crop-specific terms may reuse the same trait name, the specific crop's common name is added as a prefix to the existing CO term name to disambiguate them on the Planteome browser (Figure 1C).

Mapping the CO crop-specific ontologies to the Trait Ontology allows inter-specific comparison and unifies the search of data that are collected in different contexts. At the time of the previous Planteome publication in 2018, eight crop-specific ontologies were mapped to the TO and integrated into the Planteome ontology browser: cassava (*Manihot esculenta*), lentil (*Lens culinaris*), maize (*Zea mays*), pigeon pea (*Cajanus cajan*), rice (*Oryza sativa*), soybean (*Glycine max*), sweet potato (*Ipomoea batatas*) and wheat (*Triticum aestivum*). Since that time, we have added sorghum (*Sorghum bicolor*), yam (*Dioscorea*sp.), and potato (*Solanum tuberosum*), bringing the total number to 11 (Table 2). The CO-TO mappings are maintained on GitHub along with the CO crop-specific ontologies.

Maintaining ontologies, metadata, and the inter-ontology mappings can be challenging since ontologies continue to evolve. It requires funding for ontology maintenance and development tasks, which are resource-intensive in terms of time, expertise, and computational resources. To centralize resources and provide a common cyberinfrastructure platform for ontology development for plant biology, we collaborated with partners and introduced the Planteome GitHub platform. Each CO ontology development group has read and write access to its own Planteome GitHub repository, which provides long-term storage and tools for tracking edited versions, ticketed requests, and public and development release versions of the ontology files and scripts. A Zenodo-enabled DOI is assigned to the public release version of each vocabulary integrated into the TO for easy citation and to meet FAIR data standards. Both the CO and reference ontology developers use the Planteome GitHub infrastructure.

Integrating the CO vocabularies into the TO is an involved process. Over the past several years, we have gradually moved from manual mappings where a trained curator maps the CO traits to a term from the TO to a more automated system using ontology design patterns (DP; Table 2) (21). Even though the current automated mapping techniques streamline the maintenance process, human intervention, and continuous monitoring are still essential to ensure the quality and accuracy of ontology mappings over time. Our approach for guaranteeing the long-term sustainability of the mappings between the crop-specific CO traits and the reference TO also encourages community engagement. It allows quick turnaround in building ontology updates and helps correct deficiencies and inconsistencies in reference ontologies and the CO vocabularies.

A first test release of a pilot program of automated mapping and community engagement is underway, where we reuse the Simple Standard for Sharing Ontology Mappings (SSSOM) (22) format to generate and store the mappings. The SSSOM enables machine-readable mappings and the opportunity for community input and edits. While design patterns were used earlier for mapping, we are now testing mapping generation using an in-house SSSOM-based lexical matching algorithm. We use SKOS (23) properties to categorize the mappings, namely exact (skos:exactMatch) or subclass (skos:broadMatch or skos:narrowMatch). During this process, we also attach metadata to the mappings, including the type of match (e.g. HumanCurated, lexical) and provenance information, such as the version of the ontologies used to generate the mappings. These mappings are publicly available in a tabular format in the respective GitHub repositories, making it easy for anyone to contribute to their creation and curation. This approach was tested for rice, sorghum, and potato CO vocabularies.

### New tools

#### Planteome-CO term request form

**Figure 2.**
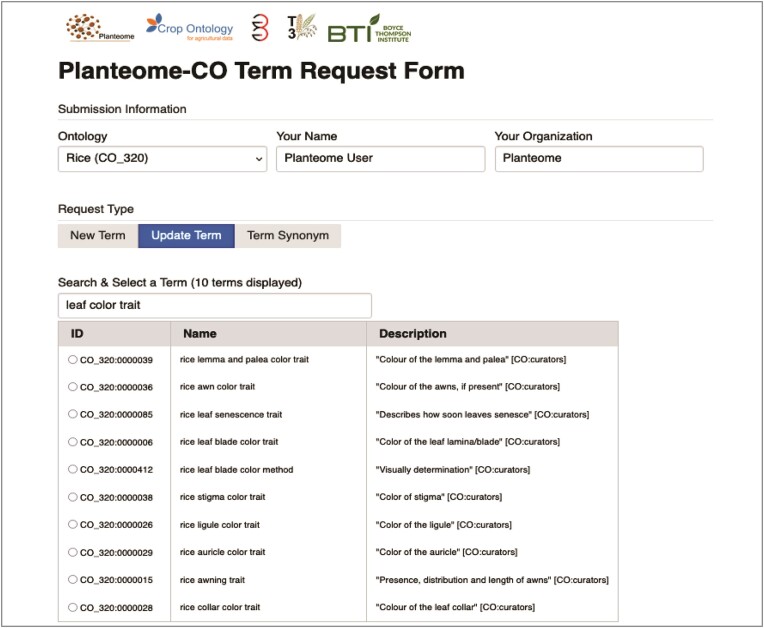
Planteome-CO term request form. Curators or ontology users can submit requests or suggest changes to any 29 Crop Ontologies (CO) hosted on the Planteome GitHub. If one of the eleven CO integrated into the Planteome is selected, the ten closest term matches are displayed when 'Update Term' is selected.

We implemented an online Planteome-CO term request form (https://trait-requests.planteome.org/) to engage community participation and simplify user requests for new ontology terms, suggestions for potential edits, or to add synonyms to existing terms (Figure 2). Requests can be submitted using a GitHub ID, or by simply supplying an email address in case requesters are not familiar with GitHub.

The term request form is a collaborative effort between Planteome, the CO, GrainGenes (https://wheat.pw.usda.gov/GG3/), the Triticeae Toolbox (https://triticeaetoolbox.org/), and the CGIAR program on roots, tubers and bananas (https://www.rtb.cgiar.org/). In the backend, the requests made through this tool are linked to the ticketing issue tracker for the respective Planteome GitHub repository. Upon submission, the tool automatically generates a GitHub issue ticket, alerts the curator, and allows the requester to track the progress and resolution. Currently, the Term Request tool links to 29 CO vocabularies on GitHub and includes the 11 integrated into the Planteome knowledgebase and described above. If users wish to suggest a new trait or request an edit to an existing term, they can select the appropriate button and fill in the corresponding boxes. If one of the linked CO vocabularies is selected, a drop-down list of 10 of the most closely related existing terms is displayed, along with the definition, if one exists (Figure 2).

To request a new term, the user should fill in the ‘Trait Name’ box, select an appropriate ‘Trait Class’ from the drop-down menu, and provide a detailed definition and any relevant synonyms. If requesting an update to an existing term (linked CO only), the user can enter a term name or identifier (e.g. CO_320:0000146) and select from the list of terms shown. The user should fill in the details of the requested change and its rationale.

#### Plant Ontology glossary

**Figure 3.**
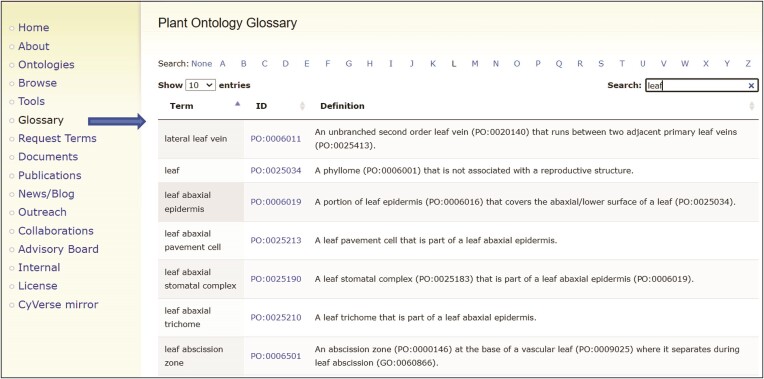
Plant Ontology glossary. Plant Ontology terms in the most recent release can be browsed and searched quickly in the interactive glossary format. The term name, PO identifier and definition are displayed. The PO identifier hyperlinks lead to the ontology browser.

Another new feature for our users is the interactive plant ontology glossary (https://planteome.org/po_glossary) (Figure 3). All the terms in the current release version of the PO are browsable and searchable (including obsolete terms). The full term name is displayed along with the identifier and definition. The user can browse the ontology by alphabetical letter and filter the results using the ‘Search’ box. The results can also be sorted by term name or identifier using the arrow buttons above the list. Users can access the PO Glossary from the menu link on the left-hand side of the Planteome home page.

#### Planteome knowledge graph

A knowledge graph (KG) is a graph-based data model (24) synthesized by building connections among diverse data types (nodes) and interdependencies (edges) for intra- and interspecies comparisons of genes, pathways, phenotypes and experimental data. Additional data nodes and edges in the graph may include natural and synthetic genetic variation and its functional consequences on gene function, expression, and phenotype to support researchers in novel hypothesis generation for translational research and advancing knowledge. For a database such as Planteome, managing the continuous deluge of multidimensional data depends on cleaning, formatting, analyzing, mapping to entities (and different versions) in multiple online databases, routine updates, and remotely accessing data APIs. The process is error-prone and inefficient, restricts data usage by researchers, and almost always requires them to perform additional analyses to synthesize the knowledge. Researchers find it difficult to access data from multiple sources often provided in different formats. There are many data repositories and databases; however, we need knowledge graphs built on the prior knowledge collected from multiple sources and a sustainable mechanism to provide new versions with new and enriched data layers. When implementing additional deep learning tools on these KGs, one may be able to create subgraphs with pieces of information that are not previously curated or generate new hypotheses to test.

To empower researchers, Planteome developed a pilot knowledge graph (25), based on the Biolink model (24), which incorporates ontology terms and annotation data to allow users to make complex queries and help discover novel genotype-phenotype or molecular interactions. Using the KG, researchers can start with their queries using the graph to navigate the current data and utilize the power of deep learning to synthesize knowledge, find dependencies, build hypotheses, and find deficiencies. The Planteome KG integrates ontologies and annotation data from Planteome with experimental data from the EMBL-EBI Gene Expression Atlas. Therefore, the Planteome KG connects gene expression, molecular interactions, functions, and pathways to homology-based gene annotations and includes environmental conditions or treatments (E) that allow genotype (G) to be linked to phenotype (P) as G2P or GxE = P. Planteome considers gene expression a form of molecular-level phenotype often regulated by the environment and the plant's spatial and temporal landmarks.

#### Future directions

The future directions of the Planteome include developing the Plant Stress Ontology, which will link together phenotype traits, biotic and abiotic causal agents of plant stress, the associated plant structures, and growth stages that the stress may (or is known to) affect, and the experimental conditions. New CO vocabularies for banana, groundnut, oat, and cowpea will be mapped to the reference TO and integrated into the Planteome knowledgebase. The SSSOM pilot project will be further fine-tuned and expanded to include all the Crop Ontology into Planteome. To maintain the accuracy and completeness of the mappings over time following the different ontology updates, we plan on utilizing GitHub actions to sync the mappings with the ontologies autonomously. Any update in the ontologies will automatically trigger GitHub actions to perform the re-mappings, update the SSSOM file storing the mappings, and notify the community and curators that new mappings need curation or review. We plan to scale up the knowledge graph development to include the nascent plant stress ontology and to implement futuristic applications of Large Language Models and OntoGPT frameworks (26,27) to enable this process, besides helping improve annotation deficiencies and human curation overload.

## Data Availability

The data and resources above are available at the main project website, https://planteome.org, and licensed under a Creative Commons Attribution 4.0 International License. The ontology browser is available at https://browser.planteome.org or by clicking the links labeled "Browse" or "Ontology Browser" on the project web site. The Planteome ontologies are maintained in their respective ontology-specific repositories in the Planteome GitHub organization (https://github.com/Planteome). The annotation files are too large to be stored at GitHub and so are stored on our own internal Subversion Repository at https://palea.cgrb.oregonstate.edu/viewsvn/associations/.
